# Effect of ultrasound-assisted sodium bicarbonate treatment on gel characteristics and water migration of reduced-salt pork batters

**DOI:** 10.1016/j.ultsonch.2022.106150

**Published:** 2022-08-30

**Authors:** Zhuang-Li Kang, Xue-Yan Shang, Yan-Ping Li, Han-Jun Ma

**Affiliations:** School of Food Science, Henan Institute of Science and Technology, Xinxiang 453003, PR China

**Keywords:** Ultrasound, Sodium bicarbonate, Relaxation time, Textural properties, Microstructure

## Abstract

To study the potential usefulness of ultrasound (0, 30, and 60 min) and sodium bicarbonate (0 % and 0.2 %) combination on the reduced-salt pork batters, the changes in water holding capacity, gel properties, and microstructure were investigated. The pH, salt-soluble proteins solubility, cooking yield, and b* values of reduced-salt pork batters significantly increased (*P* < 0.05) with the increase in ultrasound time and the addition of sodium bicarbonate, leading to the hardness, springiness, cohesiveness, and chewiness significantly increased (*P* < 0.05). Furthermore, the use of ultrasound-assisted sodium bicarbonate treatment caused the reduced-salt pork batters to form a typical spongy structure with more evenly cavities. Due to the initial relaxation time of T_21_ and T_22_ were shorter, and the peak ratio of P_21_ was increased and P_22_ was decreased after ultrasound-assisted sodium bicarbonate treatment, implying that the mobility of water was reduced. Thus, the use of ultrasound-assisted sodium bicarbonate treatment enabled reduced-salt pork batters to have better gel characteristics and higher cooking yield.

## Highlights

• Ultrasound-assisted NaHCO_3_ increased pH, SSP, cooking yield of reduced-salt batter.
• Ultrasound-assisted NaHCO_3_ improved the texture and microstructure of batters.
• The mobility of water was reduced after treated with ultrasound-assisted NaHCO_3_.
• Ultrasound-assisted NaHCO_3_ enabled reduced-salt batters to have good gel properties.

## Graphical abstract

**Figure:**
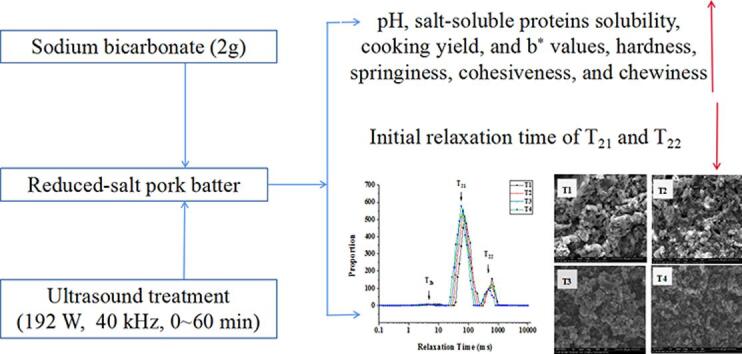

## 1 Introduction

Currently, due to consuming too much sodium chloride can increase blood pressure, and it is the leading cause of death and disability among adults in the world [10], [7], more and more consumers pay attention to the reduced-salt meat products. However, because sodium chloride improves the functional properties of the myofibrillar protein, enhances flavour and inhibits microorganisms, it determines their colour, cooking yield, texture properties, taste and shelf life [4], [42]. When the sodium chloride content is <0.3 mol/L, the swelling of myofibrillar protein is decreased, leading to the functional properties of gel meat products being lowered [41]. So reducing sodium chloride in gel meat products is difficult.

Ultrasound as a new technology has great development potential and broad application prospects, it has the advantages of high safety, green environmental protection, low operating cost and instantaneous efficiency compared with other processing methods, and become an important research field in the reduced-salt meat products, such as accelerating the brining process, mass transfer, curing process and reduction cooking time [14], [27], [6], [2], [17]. Kang et al. [19] reported that the ultrasound treatment can modify the structure and thereby improve the functional properties of myofibrillar protein during meat processing, leading to quality enhancement, low fat and/or salt products development and the shelf life-extending. Sodium bicarbonate as a common phosphorus-free food additive can induce alkaline conditions in water, and improve the protein solubility and water retention ability by increasing the pH value and ionic strength of meat [36], [48]. Our previous study found that the moderate addition of sodium bicarbonate could improve the overall palatability of meat products with better water retention and texture properties, thus effectively improving the quality of pork meat batters [19]. Some studies have shown that the use of ultrasound-assisted sodium bicarbonate treatment can improve the chicken breast meat tenderization, water holding capacity and curing efficiency by increasing the myofibril fragmentation indexes (MFI), the interfibrillar spaces and the degree of actomyosin dissociation, causing the conformation change in actomyosin [43], [49]. However, to our knowledge, there remain large unknowns that the gel properties and water holding capacity of reduced-salt pork batters were affected by ultrasound-assisted sodium bicarbonate treatment. Based on this, we hypothesized that ultrasound-assisted sodium bicarbonate treatment can enhance the pH and salt-soluble proteins (SSP) solubility of reduced-salt pork batters, leading to the texture properties and water holding capacity improved. Therefore, the objective of this study was to investigate the effect of ultrasound-assisted (0, 30, and 60 min) sodium bicarbonate (0 % and 0.2 %) on the gel properties, water distribution and microstructure of reduced-salt pork batters, find a novel method to improve the quality of reduced-salt meat batters.

## 2 Materials and methods

### 2.1 Materials

Chilled pork [*Duroc*×*(Landrace* × *Yorkshire)*, (180 ± 3 d old, 100 ± 5 kg)] lean leg meat (*mesoglutaeus*; after slaughter 24 h-48 h, central temperature 2 ± 2 °C; pH 5.65 ± 0.01; protein 20.37 ± 0.51 %) and pork back-fat (89.63 ± 0.81 %) were provided by a locate slaughterhouse (Xinxiang, China). Removing the visible fat and connective tissue, the pork meat was ground with a 6 mm hole plate using a meat grinder (JR-120, Shandong, China) and mixed uniformly. Following, approximately 200 g of ground meat was vacuum packaged in a nylon/PE bag and stored at −20 °C within 30 d. Sodium bicarbonate and sodium chloride (analytically pure) were supplied by Tianjin Boddi Chemical Co., ltd., China. The spices were purchased from a local market (Xinxiang, China).

### 2.2 Preparation of the pork batters and ultrasound treatment

The pork batter was performed according to the method of Kang et al. [21]. The formulas of raw batters were as follows: pork meat 1000 g, pork back-fact 200 g, ice water 200 g, spices 13.5 g, therein, T1 contained sodium chloride 25 g, sodium bicarbonate 0 g; T2, T3, and T4 contained sodium chloride 20 g, sodium bicarbonate 2 g. Then the batter was vacuum packaged for ultrasonic-treated using an ultrasonic machine (SB-4200, Ningbo Xinzhi Biotechnology Co., ltd., China) at 4 ± 2 °C. According to the method of Li et al. [26] and modification, the ultrasound conditions were as follows: the power was 192 W, the frequency was 40 kHz, therein, the ultrasound time of T1 and T2 was 0 min, T3 was 30 min, and T4 was 60 min. After that, the batter was heated at 80 °C using a water bath for 20 min (core temperature was 72 °C), then cooled to 20 °C using running water and stored at 2 ± 2 °C; the other was stored at 2 ± 2 °C for measuring pH, cooking yield, and SSP solubility.

### 2.3 pH

Approximately 10 g of each raw pork batter was mixed with 40 mL of distilled water (4 °C) homogenized (High-speed homogenizer, Ningbo Xinzhi Biotechnology Co., ltd, China) at 15000 rpm for 10 s in an ice bath. And then the pH was measured by a digital pH meter (PHS-2F, Shanghai Electrical Instrument Co., ltd., China).

### 2.4 SSP solubility

SSP solubility was determined according to the method of Cofrades et al. [11]. Approximately 10 g of raw pork batter was mixed with 50 mL 20 mM phosphate buffer (0.6 M NaCl, 2–4 °C, pH 7.0), homogenized at 15000 rpm in an ice bath (High-speed homogenizer, Ningbo Xinzhi Biotechnology Co., ltd., China), and then centrifuged at 8000×*g* for 30 min (225 High-speed frozen centrifuges, Fischer Test Instruments ltd., Germany). The protein content was determined according to Lowry, Rosebrough, Farr and Randall [31], using bovine serum albumin as a standard.

### 2.5 Cooking yield

The pork batter was placed at 2 ± 2 °C storage overnight, put on the surface of water before being measured. Cooking yield was calculated according to the formula:

Cooking yield (%) = Weight of cooked batter/Weight of raw batter × 100 %.

### 2.6 Texture profile analysis (TPA)

The texture properties of cooked pork batter were measured according to Zhu et al. [47]. The batter was removed to the laboratory at 20 °C and left for 2 h, then it was cut into a cylinder with a diameter was 15 mm and a height was 15 mm. The TPA of batter was determined using a texture analyzer with a P/36 R probe (TA-XT.plus, Stable Micro system ltd., Surrey, UK). The setting parameters were as follows: pre-test speed 5.0 mm/s; test speed 2.0 mm/s; post-test speed 2.0 mm/s; strain 50 %, time 5.0 s; and trigger force 5 g. The hardness (N), springiness, cohesiveness and chewiness (N·mm) of the batter were obtained.

### 2.7 Colour

The center colour of the cooked pork batter was determined using a colourimeter (CR-400, Minolta Camera Co., Japan) with a pulse xenon lamp (the aperture is a diameter of 11 mm), calibrated with a standard white plate (L* = 96.86, a* = −0.15, b* = 1.87). Six fresh slices from each batter were evaluated within 1 min.

### 2.8 Low field nuclear magnetic resonance (LF NMR)

According to the method of Kang et al. [24], the NMR relaxation measurements were measured by an NMI20-040V-I low-field NMR analyzer (Niumag Electric Corporation, Shanghai, China). Approximately 2 g cubes of cooked batter was cut into and packaged in Ziplock bags (PE), then left at a 32 °C thermostat for 30 min. The setting parameters were as follows: value set to 200 s, repeated scan 32 times, get 12,000 echoes, temperature 32 °C. The post-processing of the NMR T2 data distribution involved the exponential fitting of CPMG (Carr-Purcell-Meiboom-Gill) decay curves and it was implemented by using Multi-Exp Inv Analysis software (Niumag Electric Corp, Shanghai, China).

### 2.9 Microstructure

The microstructure was measured by scanning electron microscopy (Hitachi-S-800, Hitachi High Technologies Corp., Tokyo, Japan), referred to as the method of Haga and Ohashi [15].

### 2.10 Statistical analysis

The entire experiment was repeated four times at different times using different source materials (sodium bicarbonate and sodium chloride) and ultrasound times (0, 30, and 60 min). Results were expressed as the mean ± SE. The data were analyzed using the statistical software package SPSS v.0.26.0 (SPSS Inc., Chicago, USA). Data were analyzed through the general linear model (GLM) procedure, considering the treatments (sodium bicarbonate concentration and ultrasound time) as a fixed effect and the replicates as a random effect. Significant differences between means were identified by the LSD procedure. The difference between means was considered significant at *P* < 0.05.

## 3 Results and discussion

### 3.1 pH

Effects of ultrasound-assisted sodium bicarbonate treatment on the pH of raw pork batter are shown in Table 1. The pH of raw pork batter significantly increased (*P* < 0.05) when the sodium bicarbonate was added. The reason is that sodium bicarbonate is a strong alkali and weak acid salt, it can produce the bicarbonate ions after solubles in water and makes the water alkaline, thus, the pH of raw pork batter could be increased [32], [48]. Kang et al. [19] reported that the pH of low-salt pork batter was significantly increased with the increase of sodium bicarbonate from 0 % to 0.42 %. A similar study has shown that the pH of raw chicken batter with 0.5 % sodium bicarbonate was increased by approximately 0.4 units compared with the sample without sodium bicarbonate [47]. Meanwhile, the pH of raw pork batter significantly increased (*P* < 0.05) with the increase in ultrasound time, except for the samples of T1 and T2, T2 and T3. The reason is possible that the release of ions from the cellular structure into the cytoplasm, and the changes in the protein structure, which results in a modification of the position of some ionic groups, allows them to be used for muscle buffer reaction [1]. A previous study has shown that the pH of beef elevates by nearly 0.05 units after ultrasonic treatment (40 kHz, 60 min) (Peña-Gonzalez et al., 2019). Jayasooriya et al [18] found that the pH of bovine *Semitendinosus* and *Longissimus* muscles improves after ultrasonic treatment (24 kHz, 240 s). Thus, the use of ultrasound-assisted sodium bicarbonate treatment could improve the pH of raw pork batter than the ultrasound treatment alone.

**Table 1:** Effects of ultrasound-assisted sodium bicarbonate treatment on the pH, cooking yield (%), and salt-soluble proteins solubility (%) of raw pork batters.

Sample	pH	Salt-soluble proteins solubility (%)	Cooking yield (%)
T1	5.68 ± 0.03^d^	30.30 ± 0.61^d^	86.74 ± 0.63^d^
T2	5.91 ± 0.02^c^	37.23 ± 0.61^c^	91.22 ± 0.19^c^
T3	5.97 ± 0.03^b^	39.50 ± 0.60^b^	92.97 ± 0.39^b^
T4	6.05 ± 0.03a	43.23 ± 0.38a	94.79 ± 0.95a

T1, sodium bicarbonate 0 g, ultrasound time 0 min; T2, sodium bicarbonate 2 g, ultrasound time 0 min; T3, sodium bicarbonate 2 g, ultrasound time 30 min; T4, sodium bicarbonate 2 g, ultrasound time 60 min.

Each value represents the mean ± SE, n = 4.

a–d different parameter superscripts indicate significant differences(*P* < 0.05).

### 3.2 SSP solubility

Effects of ultrasound-assisted sodium bicarbonate treatment on the SSP solubility of raw pork batter are shown in Table 1. The SSP solubility of raw pork batter significantly increased (*P* < 0.05) when the sodium bicarbonate was added. The reason is that the pH was increased with the addition of sodium bicarbonate (Table 1), leading to the SSP shifts away from the isoelectric point and promoting the dissolution of the protein [37], [19] (Lee et al., 2015). Some researchers have shown that the addition of sodium bicarbonate prompts the myofibrillar proteins to dissolve, forms a clear solution with the smaller aggregates, and decreases turbidity [29], [35]. In addition, the SSP solubility of raw pork batters significantly increased (*P* < 0.05) with the increase in ultrasound time. Besides the reason is that ultrasound treatment gradually increased pH (Table 1), the other reason is possible that ultrasound treatment leads to physical disruption of muscle tissue and accelerated mass transfer through cavitation-related mechanisms, which significantly improved myofibrillin breakage, solubility and leads to the disintegration of connective tissue in meat [43], [8], which improve the extraction of SSP [30]. So the use of ultrasound-assisted sodium bicarbonate treatment could improve the SSP solubility of raw pork batter by shifting the pH and breaking the myofiber.

### 3.3 Cooking yield

The changes in the cooking yield of raw pork batter treated with ultrasound-assisted sodium bicarbonate are shown in Table 1. The cooking yield of the pork batter significantly increased (*P* < 0.05) with the increase in ultrasound time and the addition of sodium bicarbonate. The result was in agreement with the result of SSP solubility (Table 1). Some studies have reported that adding sodium bicarbonate in moderation can increase the water- and fat-holding capacities during the processing, and decrease the cooking loss of pork batters [19], chicken batters [32], [47], ground beef [33]. Moreover, because the increase in SSP solubility after ultrasound treatment, increases the ability of the pork batter to prevent water from spreading outside [9], [28]. Xiong et al. [43] found a similar result is that the cooking yield of chicken breast improved after ultrasound-assisted sodium bicarbonate treatment (20 kHz, 300 W; 10 min; 4 °C), due to the ultrasonic cavitation can promote the penetration of sodium bicarbonate solution into the tissue and destroy the myofibrillary structure of meat [49].

### 3.4 Texture properties

TPA is a double compression test is used to judge the texture properties of food and link them to sensory properties. The changes in texture properties of cooked pork batter treated with ultrasound-assisted sodium bicarbonate are shown in Table 2. Compare with the sample of T1, the hardness, springiness, cohesiveness and chewiness of cooked pork batter significantly increased (*P* < 0.05) with the increase in ultrasound time and the addition of sodium bicarbonate. The result was in agreement with the result of SSP solubility (Table 1). It is well known that SSP solubility is an important impact on the textural properties of meat products [48], [22]. The reason is that more proteins were unfolded and more aliphatic residues were exposed when the increase in SSP solubility, which caused of more the sites in the polypeptide chains were crosslinking and form more protein aggregations before the heating, leading to a stable, elastic and rigid gel structure is formed during the heating [20], [40]). Previous studies have reported that added sodium bicarbonate improves the texture properties of emulsion meat products, nevertheless, too much sodium bicarbonate can produce more carbon dioxide and destroy the gel structure, then cause the texture properties to decrease [19], [47]. In addition, the cavitation effect of ultrasound can increase the solubility of the myofibrillar protein, which favour the formation of gel structure [45]. Moreover, some researchers found that the content of β-sheet structure of cooked batter is significantly increased and accompanied by the content of α-helice structure is significantly decreased when the increase in ultrasound time and the addition of sodium bicarbonate [26], [47], due to the β-sheet structure is the basis of gel, which can form a stable, elastic and rigid gel matrix.

**Table 2:** Effects of ultrasound-assisted sodium bicarbonate treatment on the texture properties of cooked pork batters.

Sample	Hardness (N)	Springiness	Cohesiveness	Chewiness (N.mm)
T1	49.75 ± 0.64d	0.845 ± 0.009d	0.627 ± 0.004d	28.96 ± 0.88d
T2	56.80 ± 0.66c	0.902 ± 0.009c	0.692 ± 0.006c	38.54 ± 0.42c
T3	58.26 ± 0.74b	0.918 ± 0.005b	0.708 ± 0.007b	40.26 ± 0.63b
T4	60.79 ± 0.38a	0.931 ± 0.005a	0.724 ± 0.006a	42.73 ± 0.32a

T1, sodium bicarbonate 0 g, ultrasound time 0 min; T2, sodium bicarbonate 2 g, ultrasound time 0 min; T3, sodium bicarbonate 2 g, ultrasound time 30 min; T4, sodium bicarbonate 2 g, ultrasound time 60 min.

Each value represents the mean ± SE, n = 4.

a–d different parameter superscripts indicate significant differences (*P* < 0.05).

### 3.5 Colour

Colour is a key factor in meat quality because it is the first sensory feature of consumer evaluation. Consumers take the colour of meat products as one of the important criteria for judging (Peña-Gonzalez et al., 2018). As shown in Table 3, ultrasound-assisted sodium bicarbonate treatment has a significant effect on the colour of pork batter. Compare with the sample of T1, the L* value of cooked pork batter significantly increased (*P* < 0.05) when the addition of sodium bicarbonate, but it did not significantly different (*P* > 0.05) with the increase in ultrasound time; the a* value significantly decreased (*P* < 0.05) with the increase in ultrasound time and the addition of sodium bicarbonate, on the contrary, the b* value significantly increased (*P* < 0.05) with the increase in ultrasound time and the addition of sodium bicarbonate. It is well known that shifted pH can lower the oxidation rate of myoglobin to metmyoglobin [25], thus, the a* value of cooked pork batter was decreased, and the b* value was increased after ultrasound-assisted sodium bicarbonate treatment. Previous studies have reported that increasing sodium bicarbonate while decreasing sodium chloride did not affect the L* values of cooked normal pork batters, the a* values were decreased, and the b* values were increased [19], [33]. These differences are caused by the different processing methods. Fallavena et al. [12] reported that due to the ultrasound treatment (20 kHz, 84 W) promotes the formation of oxidized myoglobin and slows down the formation of high iron myoglobin, which makes the beef indicate red less prominent and strong yellow.

**Table 3:** Effects of ultrasound-assisted sodium bicarbonate treatment on the colour of cooked pork batters.

Sample	L* value	a* value	b* value
T1	75.05 ± 0.30b	4.90 ± 0.06a	10.17 ± 0.12d
T2	76.33 ± 0.35a	3.88 ± 0.04b	11.03 ± 0.19c
T3	76.46 ± 0.40a	3.47 ± 0.13c	12.44 ± 0.07b
T4	76.66 ± 0.22a	3.17 ± 0.04d	13.24 ± 0.27a

T1, sodium bicarbonate 0 g, ultrasound time 0 min; T2, sodium bicarbonate 2 g, ultrasound time 0 min; T3, sodium bicarbonate 2 g, ultrasound time 30 min; T4, sodium bicarbonate 2 g, ultrasound time 60 min.

Each value represents the mean ± SE, n = 4.

a–d different parameter superscripts indicate significant differences(*P* < 0.05).

### 3.6 LF NMR measurements

**Fig. 1:**
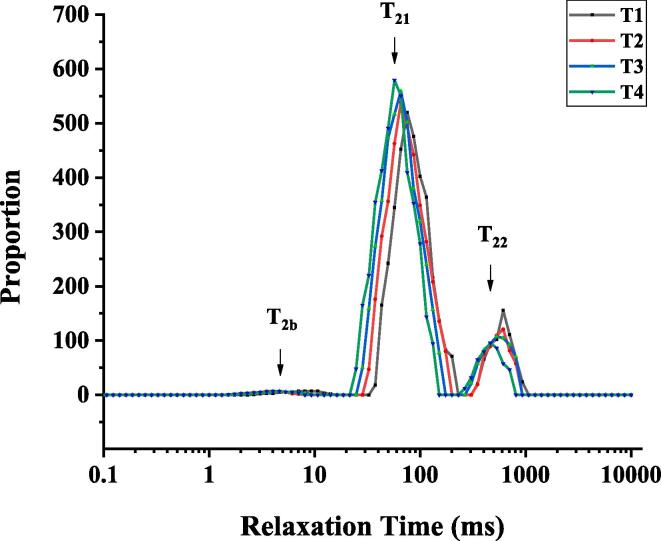
Effects of ultrasound-assisted sodium bicarbonate treatment the relaxation time (ms) of cooked pork batters. T1, sodium bicarbonate 0 g, ultrasound time 0 min; T2, sodium bicarbonate 2 g, ultrasound time 0 min; T3, sodium bicarbonate 2 g, ultrasound time 30 min; T4, sodium bicarbonate 2 g, ultrasound time 60 min.

Low-field NMR can reflect the holding water situation of the pork batter, especially the proton transverse relaxation time, which can be used to evaluate the water distribution and mobility inside the gel [19], [16]. The changes in proton transverse relaxation time of cooked batter treated with ultrasound-assisted sodium bicarbonate are shown in Fig. 1. The peaks of T_2b_, T_21_ and T_22_ were observed in the inversion map of nuclear magnetic intensity, and they were located in 0.01–10 ms, 10–100 ms, and 100–1000 ms, respectively [14], [36]. Therein, T_2b_ represents the bound water, it is tightly adsorbed and bound to protein and macromolecular constituents; T_21_ represents the immobile water, It depends on the network structure of the myofibrillary protein and how much static charge the protein carries; T_22_ represents the free water, it mainly exists in gel structure by capillary force and loosely bound in sol matrix of meat batters [5].

In this study, the changes in the initial relaxation time and peak ratio of cooked pork batters treated with ultrasound-assisted sodium bicarbonate are shown in Table and Fig. 1. The initial relaxation time of T_2b_ decreased significantly (*P* < 0.05) when the addition of sodium bicarbonate, and they did not significantly different (*P* > 0.05) with the increase in ultrasound time, implying that the bound water from cooked batters with sodium bicarbonate was tied closer than the sodium chloride [43], [44], while the effect of adding sodium bicarbonate on the binding water was greater than that of ultrasound treatment. Li et al [26] reported that the T_2b_ of reduced-salt chicken breast meat batter was not significantly different (*P* > 0.05) with the increase in ultrasound time. The initial relaxation time of T_21_ and T_22_ decreased significantly (*P* < 0.05) after adding sodium bicarbonate, and they increased significantly (*P* < 0.05) with the increase in ultrasound time (Table 4). The possible reason was that the use of ultrasound-assisted sodium bicarbonate treatment can improve the water holding capacity and texture properties (Table 1, Table 2). Meanwhile, all the peak ratios of P_2b_ did not significantly different (*P* > 0.05), and the peak ratio of *P*_21_ increased significantly (*P* < 0.05) with the increase in ultrasound time and the addition of sodium bicarbonate, on the contrary, the peak ratio of P_22_ decreased significantly (*P* < 0.05). The results were consistent with the initial relaxation time of cooked pork batters (Table 4). The reason is that the use of ultrasound-assisted sodium bicarbonate treatment could increase the pH and SSP solubility, and improve the texture properties, leading to the water holding capacity being enhanced.

**Table 4:** Effects of ultrasound-assisted sodium bicarbonate treatment on the initial relaxation time (ms) and peak ratio (%) of cooked pork batters.

Sample	Initial relaxation time (ms)			Peak ratio (%)		
	T_2b_	T_21_	T_22_	P_2b_	*P*_21_	P_22_
T1	2.51 ± 0.17^a^	35.23 ± 1.18^a^	373.26 ± 11.65^a^	1.23 ± 0.19^a^	86.37 ± 0.66^d^	12.53 ± 0.42^a^
T2	1.82 ± 0.15^b^	32.07 ± 1.08^b^	346.20 ± 12.22^b^	1.17 ± 0.27^a^	89.25 ± 0.81^c^	9.31 ± 0.29^b^
T3	1.67 ± 0.19^b^	28.81 ± 1.35^c^	308.85 ± 10.84^c^	1.11 ± 0.20^a^	91.50 ± 0.73^b^	7.93 ± 0.36^c^
T4	1.61 ± 0.15^b^	25.52 ± 1.20^d^	267.23 ± 13.09^d^	0.97 ± 0.18^a^	94.66 ± 0.90^a^	5.56 ± 0.45^d^

T1, sodium bicarbonate 0 g, ultrasound time 0 min; T2, sodium bicarbonate 2 g, ultrasound time 0 min; T3, sodium bicarbonate 2 g, ultrasound time 30 min; T4, sodium bicarbonate 2 g, ultrasound time 60 min.

Each value represents the mean ± SE, n = 4.

a–d different parameter superscripts indicate significant differences (*P* < 0.05).

### 3.7 Microstructure

**Fig. 2:**
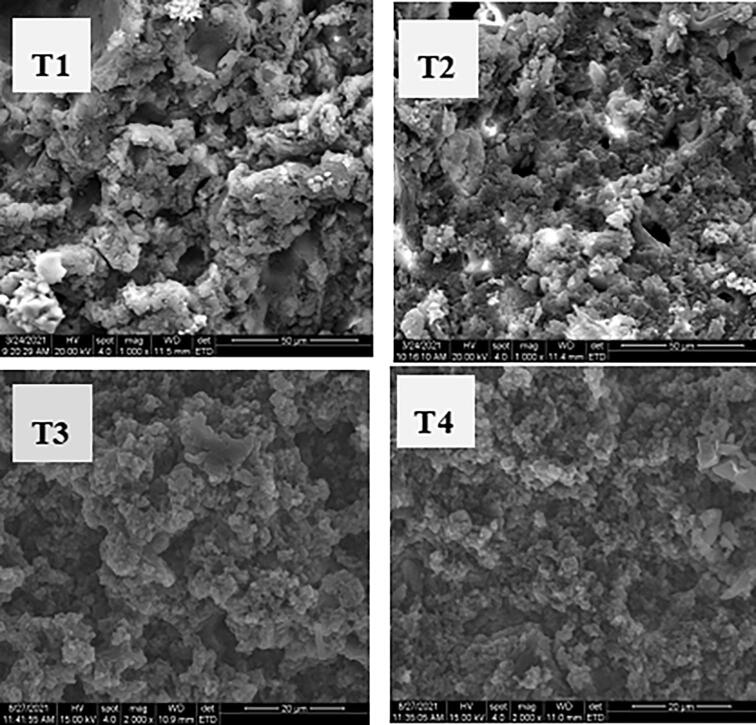
Effects of ultrasound-assisted sodium bicarbonate treatment on the microstructure of cooked pork batters. T1, sodium bicarbonate 0 g, ultrasound time 0 min; T2, sodium bicarbonate 2 g, ultrasound time 0 min; T3, sodium bicarbonate 2 g, ultrasound time 30 min; T4, sodium bicarbonate 2 g, ultrasound time 60 min.

The changes in the microstructure of cooked pork batter treated with ultrasound-assisted sodium bicarbonate are shown in Fig. 2. All treatments had a typical spongy structure of cooked meat batters, and the fat particles were surrounded [39]. Compare with the sample of T1, the samples with sodium bicarbonate had more cavities, they were caused by the production of carbon dioxide during the heating processing. Li et al. [29] reported that the absolute values of the Zeta potential, active sulfhydryl, and surface hydrophobicity increased significantly when the sodium bicarbonate was increased from 0 % to 0.4 %. Zhang et al. [46] found that ultrasound treatment can reduce the total thiol content, increase the surface hydrophobicity of myofibrillar protein, and promote the interactions between protein molecules through disulfide bonds or hydrophobic forces, resulting in a more uniform and compact meat protein network structure. Moreover, Amiri et al. [3] showed that the ultrasonic cavitation makes the particle size of the protein more fit between the structures and the pore size of the mesh structure. Due to the pH, SSP content, and texture characteristics of pork batter being improved after ultrasound-assisted sodium bicarbonate treatment, the cavities of T3 and T4 were more evenly than the sample of T2. The previous study has reported that added sodium bicarbonate can produce a large amount of carbon dioxide, and the gas expands to form air bubbles in the batters during heating [48].

## 4 Conclusion

The study showed that the use of ultrasound-assisted sodium bicarbonate treatment significantly affected the texture properties and water holding capacity of cooked pork batters. Ultrasound-assisted sodium bicarbonate treatment significantly increased the pH, SSP solubility, cooking yield, b* value, hardness, springiness, cohesiveness and chewiness. The result of LF-MNR showed that the use of ultrasound-assisted sodium bicarbonate caused the initial relaxation time of T_21_ and T_22_ to be significantly quicker, and the peak ratio of *P*_21_ was significantly increased and P_22_ was significantly decreased, the results meant that the content of immobile water in cooked pork batter was increased and accompanied by the decrease in the content of free water after ultrasound-assisted sodium bicarbonate treatment. Overall, ultrasound-assisted sodium bicarbonate treatment could improve the gel properties and water holding capacity of reduced-salt pork batters.

## CRediT authorship contribution statement

**Zhuang-Li Kang:** Conceptualization, Methodology, Validation, Supervision, Project administration, Writing – review & editing, Data curation, Visualization. **Xue-Yan Shang:** Conceptualization, Methodology, Validation, Formal analysis, Investigation, Writing – original draft. **Yan-Ping Li:** Conceptualization, Methodology, Validation, Formal analysis, Investigation, Writing – original draft. **Han-Jun Ma:** Project administration, Funding acquisition, Writing – review & editing.

## Declaration of Competing Interest

The authors declare that they have no known competing financial interests or personal relationships that could have appeared to influence the work reported in this paper.

## Data Availability

No data was used for the research described in the article.
